# Cardiovascular and metabolic effects of intensive *Hatha Yoga* training in middle-aged and older women from northern Mexico

**DOI:** 10.4103/0973-6131.60044

**Published:** 2009

**Authors:** Arnulfo Ramos-Jiménez, Rosa P Hernández-Torres, Abraham Wall-Medrano, María DJ Muñoz-Daw, Patricia V Torres-Durán, Marco A Juárez-Oropeza

**Affiliations:** Departments of Basic Sciences, Biomedical Sciences Institute, Autonomous University of Ciudad Juarez, Av. Hermanos Escobar y Plutarco Elías Calles s/n, Cd. Juárez Chih, Mexico; 1 School of Physical Education and Sport Sciences, Autonomous University of Chihuahua, Chihuahua, Chih., Mexico; 2 Biochemistry, School of Medicine, National Autonomous University of Mexico, Mexico City, Mexico

**Keywords:** Blood lipids, cardiovascular, exercise, Mexico, yoga

## Abstract

**Background:**

*Hatha Yoga* (*HY*) can be an alternative to improve physical activity in middle-aged and older women. However, conventional *HY* (*CHY*) exercising may not result in enough training stimulus to improve cardiovascular fitness. The purpose of this study was to evaluate the effect of an intensive *HY* intervention (*IHY*) on cardiovascular risk factors in middle-aged and older women from Northern Mexico.

**Materials and Methods:**

In this prospective quasiexperimental design, four middle-aged and nine older *CHY* practicing females (yoginis) were enrolled into an 11-week *IHY* program consisting of 5 sessions/week for 90 min (55 sessions). The program adherence, *asana* performance, and work intensity were assessed along the intervention. Anthropometric [body mass index (BMI), % body fat and Σ skin folds], cardiovascular fitness [maximal expired air volume (VE_max_), maximal O_2_ consumption (VO_2max_), maximal heart rate (HR_max_), systolic (BPs) and diastolic blood pressure (BPd)], biochemical [glucose, triacylglycerols (TAG), total cholesterol (TC), high-density lipoprotein cholesterol (HDL-C), and low-density lipoprotein cholesterol (LDL-C)], and dietary parameters were evaluated before and after *IHY*.

**Results:**

Daily caloric intake (~1,916 kcal/day), program adherence (~85%), and exercising skills (*asana* performance) were similar in both middle-aged and older women. The *IHY* program did not modify any anthropometric measurements. However, it increased VO_2max_ and VE_max_ and HDL-C while TAG and LDL-C remained stable in both middle-aged and older groups (*P* < 0.01).

**Conclusions:**

The proposed *IHY* program improves different cardiovascular risk factors (namely VO_2max_ and HDL-C) in middle-aged and older women.

## INTRODUCTION

Regular exercise has been proven to be effective in preventing chronic diseases by improving the quality of life in adults and older people. Nonetheless, the aging process always involves functional, physiological, and biochemical changes that reduce the elders' ability to perform daily activities, resulting in a fear to perform heavy physical exercise.[1,2] Therefore, sports and many other physical activities may be unsafe for older people, especially for those untrained, injured, or handicapped.

*Yoga* is an ancient Indian philosophy based on diverse breathing, stretching, and meditation exercises. The "physical" part of Yoga (*Hatha*) consists of several stretching and strength-building exercises of varying degree of difficulty called *asanas*. In complementary alternative medicine, *Hatha Yoga* (*HY*) has proved to reduce stress and pain (muscle and systemic).[3] *HY* interventions also help reduce body weight and blood glucose, total cholesterol (TC) and triacylglycerols (TAG), while they help to increase HDL-cholesterol (HDL-C) in patients with type 2 diabetes and coronary artery disease.[4‐6] Moreover, they improve forced vital capacity (FVC), forced expiratory volume in 1 s (FEV1), peak expiratory flow rate (PEFR) and maximum voluntary ventilation (MVV) while reducing the maximum O_2_ consumption (VO_2max_ ) in young healthy subjects.[7]

Recent reports suggest that *HY* can be a convenient alternative of physical activity in older people, because it reduces systolic blood pressure and sleep disturbances while it improves balance and shoulder's range of motion (flexion and abduction) in female elders.[8‐10] However, the effect of an intensive *HY* program in elderly has not been studied enough. Our aim was to study the effects of an intensive *HY* (*IHY*) practice over cardiovascular fitness, and anthropometric and biochemical parameters in middle-aged and older women from Northern Mexico.

## MATERIALS AND METHODS

### Subjects

The study was conducted at the YMCA center in Chihuahua, in Northern Mexico. Seventeen healthy and physically active middle-aged (43.2 ± 3.1 years) and older (62.2 ± 5.9 years) women volunteered for the study. The inclusion criteria were as follows: (1) to be healthy, (2) conventional *HY* (*CHY*) practitioners (yoginis, 90 min, three times per week) and (3) not taking any drugs that affect either energy metabolism or hormonal status. Thirteen women (four and nine, middle-aged and older respectively) complied with these criteria and finished the study. A sports physician performed a routine physical examination including an electrocardiogram to guarantee the health status of each participant, before and during the *IHY*. Each participant signed a written and informed consent, and the Ethics Committee of the Autonomous University of Chihuahua (Mexico), as stated by the Helsinki declaration, approved the study protocol.

### Experimental design

The thirteen participants were enrolled into an 11-week *IHY* program consisting of 5 sessions/week for 90 min (55 sessions) with a prospective quasiexperimental design. All initial and final evaluations were performed in a well-ventilated, temperature (~23°C)- and humidity (~40%)-controlled room. All participants attended the laboratory twice on week 1, between 07:00 and 9:00 h, after a 10- to 12-h overnight fasting, 8- to 9-h sleep, and 72-h alcohol-free period. Subjects were asked to avoid any sort of heavy physical activity 24 h before the study. On day 1, anthropometric and food consumption data were registered. On day 2, blood pressure (BP) and a forearm blood sample were taken. This day, they also completed a maximum exercise test during which the heart rate (HR), expired air volume (VE), O_2_ consumption (VO_2_ ), and CO_2_ production (VCO_2_ ) were continuously recorded. Having finished the *IHY* intervention, a general evaluation was once again performed as previously mentioned.

### Intensive *Hatha Yoga* intervention

All participants had been already practicing a *CHY* during the past 3 years prior to the study (90 min, three times per week). Their 90-min conventional routine included 30 min of a low-impact aerobic exercise, 15 min of relaxation in a *savasana* pose (corpse pose), 35-40 min of *asanas*, and 5-10 min of concluding remarks. The *IHY* intervention consisted of 5 sessions/week for 90 min for 11 weeks (55 sessions) and was conducted by a certified *HY* instructor specialized in training older people. Each *IHY* (90 min) session consisted of the following: 5 min of supine relaxation in a *savasana* pose, 5 min of dynamic warm-up exercises, and 80 min of *asanas* (yogic postures). All this included 5 min of *pranayamas* (breath-control exercises) and 10 min of meditation in a lotus pose.[11] The dynamic warm-up exercises consisted of walking around the room, joint exercises, and stretching. During the *asanas*, all participants were encouraged to stretch as fully as possible while not exceeding the limits of their comfort, paying attention to their breathing and trying to relax. No discomfort symptoms or injuries occurred during *IHY* sessions. The adherence to the program (as percentage of *IHY* sessions completed), *asana* performance and work intensity were monitored during the study. *Asana* performance was evaluated with a Likert-type scale by the trainer as follows: Very poor = 1, poor = 2, good = 3, and very good = 4. The work intensity was recorded with a telemetric heart rate monitor, twice during the program (Polar F6; Finland).

### Anthropometry and body composition

Anthropometric indicators and body fat were assessed with an anthropometric kit (Rosscraft Tom Kit, Canada) by a trained anthropometrist, following the recommendations of the International Society of Advancement in Kinanthropometry (ISAK).[12] Precision and reliability for skin folds, diameters, and body girth measurements were as follows: Technical error 6.2, 1.5, and 1.7%, and interclass correlation coefficients ≥0.98. All data were analyzed with LifeSize software, version 2.0 (Nolds Sports Scientific; Australia). Percentage of body fat (%BF) was also estimated according to ISAK standardized equations.[12]

### Dietary assessment

The subjects were asked to keep their normal dietary habits during the study. They were not taking any vitamin supplement. Nutrient intake was assessed by the 24-h recall method in three nonsequential days including one weekend. Appropriate food models, dishware, and containers were used to improve size estimation. All dietary records were coded daily, and later analyzed for energy and macronutrient composition. The Diet Balancer software, version 1.4 c (Nutridata Software Co., NY) was used; it provides food composition data from different sources including the latest Mexican food composition and USDA tables.[13]

### Cardiovascular fitness

In order to determine maximal expired air volume (VE_max_ ) and VO_2max_ , the percentages of O_2_ and CO_2_ in inhaled and exhaled air as well as minute pulmonary ventilation were measured with a gas analyzer (Sensor Medics 29n; Yorba Linda, CA). The system was calibrated before and during each test by using certified gas mixtures of known concentrations (4% CO_2_ , 16% O_2_ , and 80% N_2_ ; 26% O_2_ and 74% N_2_ ). A 3-L syringe (SensorMedics; Yorba Linda, CA) verified the flow of gases. The environmental barometric pressure was measured by a fortin-type mercurial barometer (Princo 469; USA), and the temperature and relative humidity by a mason-type hygrometer (Taylor 5522S mason hygrometer; Canada). Exhaled gases during exercise were analyzed with the breath-by-breath system (facemask system). The exercise tests were carried out on a treadmill ergometer (Body Guard T320X, CA).

VO_2max_ was determined by the exercise protocol reported by Skiner.[14] It consisted of walking at a constant and comfortable speed: The treadmill inclination began at 0% and rose in 2% increments every 3 min until the subject decided to end the test due to fatigue. VO_2max_ and maximal heart rate (HR_max_ ) were defined as the highest O_2_ consumption and heart rate values, both recorded at the end of the exercise test.

Blood pressure (BP) was taken 15 min before the exercise protocol, in a sited and relaxed position with a standardized sphygmomanometer (Riester Empire^®^N, USA).

### Biochemical analysis

A forearm blood sample was collected 15 min before the exercise protocol in a sited and relaxed position into heparinized tubes. Hematocrit, hemoglobin, glucose, and lipid determinations were analyzed within the next 30 min after blood collection. The microhematocrit was evaluated using a microcentrifuge, and the hemoglobin content was quantified by the Drabkin method.[15] Glucose, TAG, TC, and HDL-C were quantified in fresh plasma by routine enzymatic and spectrophotometric methods, following the manufacturer's instructions (Biosystem SA, Barcelona, Spain). Low-density lipoprotein cholesterol (LDL-C) was calculated with the Friedewald equation.[16] The interassay variation was less than 5% and intraassay less than 2.25% for all lipids. Blood glucose and lipid concentrations were adjusted for the changes in the plasma volume (microhematocrit).

### Statistics

Data were analyzed using the statistical program SAS system software, version 8.0. Middle-aged and older women's anthropometry, body composition, and dietary, biochemical, and cardiovascular fitness parameters were compared at initial and final time by a PROC GLM ANOVA test. Results were expressed as mean ± SD. The nominal level of statistical significance used was *P* < 0.05.

## RESULTS

Both groups (middle-aged and older women) showed the same program adherence (~85%) and *asana* performance (3 = good skills). In addition, food consumption was not different between groups, neither before and after *IHY* intervention in terms of total energy intake (~1,916 kcal/day) nor in the energy contribution (%) from protein (~17.2%), fat (~33.2%), and carbohydrates (~49.6%). The HR_max_ was ~26 beats/min higher and BMI ~3kg /m^2^ lower in middle-aged women than that observed for the older group (*P* < 0.05). Additionally, a higher work intensity during *HY* exercises (as expressed by heart rate monitoring) was observed in older women (50 ± 5 *vs* 59 ± 6 beats/min, *P* < 0.05, in middle-aged and older women, respectively).

*IHY* did not influence any of anthropometric variables and except for BMI, all other anthropometry variables were similar between middle-aged and older groups [Table 1]. *IHY* increased the VO_2max_ and VE_max_ (*P* < 0.05) in both groups. In regard to cardiovascular fitness indicators, VO_2max_ and HR_max_ were the only two initial variables that were higher in the middle-aged group (*P* < 0.05), while all the biochemical parameters were similar in both groups. After the *IHY* training, glucose, HDL-C, and TC increased in both groups (*P* < 0.05), while this *IHY* protocol did not have any effect on TAG, LDL-C, and log (TAG/HDL-C).

**Table 1:** Effects of intensive *Hatha Yoga* program in middle-aged and older women

Parameter^1^	Middle-aged (*n* = 4)		Older (*n* = 9)	
	Initial	Final	Initial	Final
Anthropometry				
Body weight (kg)	59 ± 7	60 ± 6	63 ± 7	62 ± 8
BMI (kg/m^2^)	23 ± 2^a^	23 ± 2^a^	26 ± 3^b^	26 ± 3^b^
Body fat (%)	27 ± 5	25 ± 5	29 ± 5	28 ± 5
Σ Skin folds (cm)	159 ± 35	140 ± 41	158 ± 33	143 ± 36
Cardiovascular fitness				
VO_2_ max (ml/kg/min)	31 ± 3^a^	32 ± 2^b^	23 ± 2^b^	27 ± 2^c^
VE_max_ (l/min)	55 ± 4^a^	61 ± 2^b^	46 ± 8^a^	50 ± 5^b^
HR_max_ (beats/min)	179 ± 2^a^	181 ± 6^a^	154 ± 9^b^	153 ± 18^b^
BPs (mmHg)	116 ± 7	118 ± 10	129 ± 11	124 ± 10
BPd (mmHg)	81 ± 10	83 ± 4	86 ± 7	82 ± 6
Biochemistry				
Glucose (mg/dl)	68 ± 9^a^	99 ± 9^b^	71 ± 12^a^	88 ± 21^b^
TAG (mg/dl)	102 ± 46	145 ± 21	138 ± 62	157 ± 56
HDL-C (mg/dl)	41 ± 8^a^	58 ± 12^b^	43 ± 7^a^	56 ± 9^b^
TC (mg/dl)	176 ± 22^a^	251 ± 62^b^	186 ± 34^a^	233 ± 56^b^
LDL-C (mg/dl)	147 ± 39	154 ± 61	171 ± 35	146 ± 47
log (TAG/HDL-C)	0.41 ± 0.30	0.43 ± 0.11	0.48 ± 0.21	0.43 ± 0.17

1 Values are expressed as mean ± SD, Values within a same row with different superscript letter (^a, b, c^) are statistically different at *P* < 0.05 either between groups or initial and final measurements, BMI = Body mass index, VO_2_ = O_2_ consumption, VE_max_ = Maximal expired air volume, HR_max_ = Maximal heart rate; BPs = Systolic blood pressure, BPd = Diastolic blood pressure, TAG = Triacylglycerols, HDL-C = High-density lipoprotein cholesterol, TC = Total cholesterol, LDL-C = Low-density lipoprotein cholesterol

## DISCUSSION

*HY* is a convenient alternative to enhance physical activity in elders in such way that its popularity has increased in western societies.[17,18] However, as aforementioned, we did not found any study that analyzed the effect of *IHY* on cardiovascular physical fitness; most of the studies combine it with other physical activities, and their results are inconclusive. Some studies have shown that Yoga training neither significantly affects physical nor physiological performance[19] but instead, decreases anaerobic power.[20] Other studies have shown that *Yoga* training improves the cardiac recovery index,[21] cardiovascular endurance, and anaerobic power[22] and decreases blood pressure either at rest or during exercise.[23] Other multidisciplinary programs that include *HY* have reported a clear improvement in health-related physical fitness.[24] Here, it has been demonstrated that an 11-week *IHY* program consisting of 5 sessions/week for 90 min (55 sessions), without any other significant physical activity, improves some cardiovascular risk factors in middle-aged and older women. However, given the small sample size of subjects in this study, additional research work is required in order to support these findings. Also, another possible weakness is that we did not include sedentary women as a control group for *IHY* cardiovascular effects. However, the opportunity to work with nonsedentary women lets us to control better the intensity of the Yoga asana which in turn argues on the benefits of *IHY* instead of conventional yoga protocols. From the beginning of the study, we assumed that if *IHY* works satisfactorily in physically active women (yoginis), then the results should be guaranteed in sedentary ones, as observed in other physical intervention studies.[23,25]

Aside from asanas, *HY* includes deep breathing and meditation with warm-up, stretching, and aerobic and resistance exercises,[24] and its application is commonly a part of other multidisciplinary components of the fitness programs, like nutrition interventions and lifestyle modifications. On the other hand, the contribution of the exclusive asanas on the health-related fitness parameters is not well established in the reported results.[26] In this work, with the exception of the 5-min warm-up, only the asanas (including pranayamas and meditation exercises) were administered. Diet and physical exercise intervention programs have shown to be effective in fat and weight reductions;[27] nevertheless, in programs that included *Yoga* exercises with 12 or less months, this effect was only detected on body fat.[24,28] In the present work, the exercise energy expenditure (EEE) on asanas practice was ~976 METs/week (~1,003 kcal/week), because *HY* duration was 450 min/week, and had a metabolic equivalent of 2.17 METs/min (2.23 kcal/min).[29] Although the EEE was higher than the minimal recommended to adults to lose fat or weight,[30] no changes in anthropometry variables were detected in the present study.

The lower HR_max_ and VO_2max_ values detected in the older women could be explained by the aging process.[2] On the other hand, we did not know of any studies where the effect of exclusive *HY* program on VO_2max_ and VE_max_ was analyzed, but in multidisciplinary studies where *HY* is included, these variables have increased.[24] In this study, there were small increments in VO_2max_ and VE_max_ in both groups at the end of the program. Aerobic and resistance exercise at low-to-moderate intensity (40-70% of HR_max_ ) have shown to increase the VO_2max_ in adults.[31] In this study, the increment in VO_2max_ was higher in the older women, and this different response could be caused by the stronger effort performed during the Yoga asanas (HR ~18%) and the higher attendance than the middle-aged women did (~11.2% higher).

Metabolic syndrome and hypertension are recurrent in apparently healthy older adults from Northwest Mexico.[32] However, lifestyle modifications, including eating habits, weight reduction, and physical activity programs, and among them *HY*, may decrease these diseases.[4,5] The decreased hypertension from *HY* has been supposed to be due to parasympathetic predominance, increased baroreflex sensitivity and decreased arterial tone and peripheral resistance.[7] Our healthy subjects were physically active, with BMI and body fat on normal values; nevertheless, they were prehypertensive (BPs 120-139, BPd 80-89). The physical intensity during the asanas was too low, according to the HR recorded, and it could be because only a slight trend to diminish both the BPs and BPd at the end of the program in older women was detected.

Others have reported that a high blood glucose, lipids, and lipoproteins decrease after regular yoga exercise, performed as either multiple interventions programs or yoga alone.[4,5] The reasons for these changes are not clear, but as mentioned above, the yoga exercises have not been standardized, neither the optimal duration nor intensity. The intensity of yoga exercise is considered as low or too low.[30,33] However, for people with low aerobic fitness, it has been shown to be enough training stimulus to improve or maintain health or cardiovascular fitness.[4,5,34] The subjects in our study, in spite of showing increases in HDL-C, showed increases in TC and not a significant change in TAG; for that, this *IHY* exercise without control diet does not improve the lipid parameters. According to our results, the reasonable explanation for these increased CT and TAG values was directly related to the diet, which although was in the normal range (~1,800 kcal/day) was badly balanced as observed for the macronutriments, with lightly high values for lipids and proteins and low values for carbohydrates. This type of diet is typical of Northern Mexico and has been associated with increases in TC and LDL-C.[35] Also, there was an increase in blood glucose within normal values (*P* < 0.05). This marginal increment could be due to gluconeogenesis favoring the influx of glycerol to the hepatocyte from adipocyte's TAG breakdown,[36] possibly associated with the light trend to diminish body fat as observed.

## CONCLUSIONS

An 11-week *IHY* program consisting of 5 sessions/week for 90 min (55 sessions) was found to be capable of improving cardiovascular fitness. In spite of the relatively low intensity of the yoga practice, this program increased both VO_2max_ and HDL-C [Figure 1]. The data also suggested that *HY* asanas, practiced as a systematic physical activity and conducted by an expert instructor in untrained and aging individuals, can improve health and serve as the basis for a physically active lifestyle. However, given the small sample size of subjects in this study, additional research work is required in order to support these findings.

**[Figure 1]:**
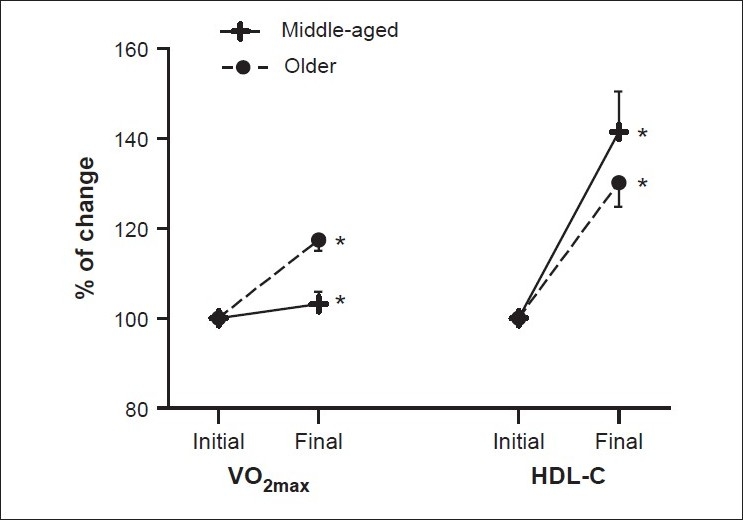
*Hatha Yoga* impacts on health fitness
